# Promotion of acceptor formation in SnO_2_ nanowires by e-beam bombardment and impacts to sensor application

**DOI:** 10.1038/srep10723

**Published:** 2015-06-01

**Authors:** Sang Sub Kim, Han Gil Na, Hyoun Woo Kim, Vadym Kulish, Ping Wu

**Affiliations:** 1 Department of Materials Science and Engineering, Inha University, Incheon 402-751, Republic of Korea; 2 Division of Materials Science and Engineering, Hanyang University, Seoul 133-791, Republic of Korea; 3 Entropic Interface Group, Singapore University of Technology & Design, Singapore 138682, Singapore

## Abstract

We have realized a p-type-like conduction in initially n-type SnO_2_ nanowires grown using a vapor-liquid-solid method. The transition was achieved by irradiating n-type SnO_2_ nanowires with a high-energy electron beam, without intentional chemical doping. The nanowires were irradiated at doses of 50 and 150 kGy, and were then used to fabricate NO_2_ gas sensors, which exhibited n-type and p-type conductivities, respectively. The tuneability of the conduction behavior is assumed to be governed by the formation of tin vacancies (under high-energy electron beam irradiation), because it is the only possible acceptor, excluding all possible defects via density functional theory (DFT) calculations. The effect of external electric fields on the defect stability was studied using DFT calculations. The measured NO_2_ sensing dynamics, including response and recovery times, were well represented by the electron-hole compensation mechanism from standard electron-hole gas equilibrium statistics. This study elucidates the charge-transport characteristics of bipolar semiconductors that underlie surface chemical reactions. The principles derived will guide the development of future SnO_2_-based electronic and electrochemical devices.

Tin oxide (SnO_2_) is an *n*-type semiconductor and is employed in a wide range of applications, including sensors for a variety of oxidizing and reducing gases. This is because it can be produced cheaply using simple methods^1^. On the other hand, sensors based on *p*-type SnO_2_ have rarely been studied. Using density functional theory (DFT) calculations for almost all possible defects, Godinho *et al.* found that the only intrinsic defects that may induce *p*-type behavior in SnO_2_ are Sn vacancies (and not interstitial oxygen as believed previously)^2^. However, owing to their high energy of formation, Sn vacancies are unlikely to form under normal conditions. While *p*-type conductivity in SnO_2_ has previously been achieved by chemically doping it with Fe^3^ or Rh^4^, in this study, we explored a physical approach for realizing *p*-type behavior in SnO_2_.

Irradiation with an electron beam is known to be an effective technique for changing or modifying materials. Irradiating electron beams has also been used under atmospheric conditions for surface modification^5^. In addition, electron-beam irradiation techniques have also been used to modify polymeric materials^6^ and fabricate power semiconductors^7^. The electron-beam irradiation reduced the Schottky barrier height of ZnO nanowire based back-to-back double Schottky diode^8^. Electron-beam irradiation of the PbO nanobelts resulted in the phase transformation from PbO_2_ to PbO and finally to Pb^9^. Edgell *et al.* reported the loss of oxygen by the electron-beam irradiation of SnO_2_ single crystals^10^. Komuro *et al.* reported the electron-beam irradiation-induced reduction of SnO_2_ to Sn on TiO_2_ surfaces^11^. The Sn-SnO_2_ coaxial nanocables were transformed to nanostructures consisting of SnO_2_ trunks and Sn branches^12^.

Synthesis techniques based on electron-beam irradiation have numerous advantages: they are simple, allow for large-scale production, involve short reaction times, and do not generate chemical residues^13^,^14^,^15^,^16^,^17^. For instance, using such a technique, silver nanoparticles have been synthesized on a large scale^13^,^14^,^15^,^16^,^17^,^18^,^19^.

In this study, we fabricated SnO_2_ nanowires and used them in gas sensors to investigate the effect of electron-beam irradiation on the sensing behavior of the nanowires. We compared the sensing characteristics of the electron-beam-irradiated SnO_2_ nanowires with those of pristine ones with respect to NO_2_ gas. Using electron-beam irradiation, we were able to achieve a transition from *n*-type sensing behavior to *p*-type behavior.

Nitrogen oxide (NO_2_) was adopted as the sensing gas not only because it is one of the gases most harmful to humans^20^ but also because NO_2_ sensors have been studied intensively in the past. One-dimensional (1D) SnO_2_ nanostructures (i.e., nanowires) were used as the sensing material, because, owing to their single crystalline structure and exceptionally high surface-to-volume ratio, they exhibit very high surface activity^21^,^22^. Finally, a network of multiple nanowires was used as the sensing scheme in the present work in order to circumvent the shortcomings associated with single-nanowire schemes^23^.

As mentioned previously, we were able to achieve a *p*-type-like semiconductor behavior in SnO_2_ nanofibers by means of electron-beam irradiation. We propose an underlying mechanism based on hole gas equilibrium statistics for the observed bipolar conduction and surface charge transport. Elucidation of the mechanism will result not only in a better understanding of charge transport in bipolar semiconductors but also lead to the development of SnO_2_-based electronic and electrochemical devices.

## Results

**Figure 1:**
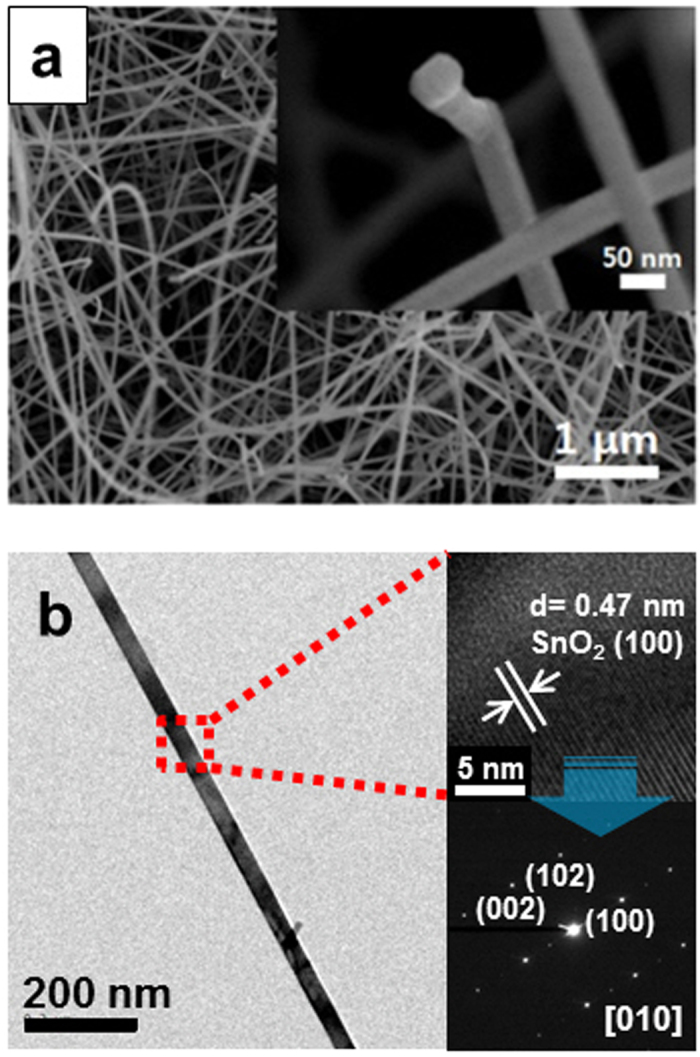
Microstructure of e-beam-irradiated SnO_2_ nanowires. (**a**) SEM image of the SnO_2_ nanowires irradiated with the electron beam at doses of 150 kGy. Insets are the corresponding high-magnification of nanowire tips. (**b**) TEM analysis of the SnO_2_ nanowires.

Figure 1a shows an SEM image of the electron-beam-irradiated SnO_2_ nanowires at doses of 150 kGy. Examination of the insets clearly reveals that no significant surface roughness was induced by the electron beam irradiation. By comparing the SEM images, it is reasonable to conclude that no significant change in the morphology and surface of the SnO_2_ nanowires resulted from electron-beam irradiation (Figure S1 in Supplementary Information). TEM analysis was performed on the electron-beam-irradiated SnO_2_ nanowires, revealing that no significant change in the crystallinity of the SnO_2_ nanowires was caused by electron-beam irradiation, Fig. 1b.

**Figure 2:**
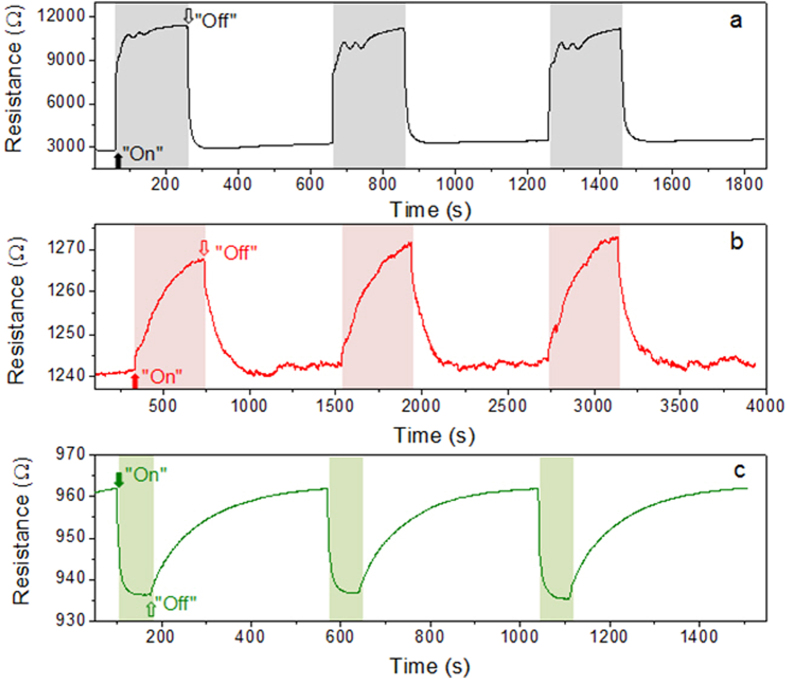
Sensing performance of e-beam-irradiated SnO_2_ nanowires. Dynamic resistance curves for the sensors fabricated from (**a**) pristine SnO_2_ nanowires and the SnO_2_ nanowires irradiated at doses of (**b**) 50 kGy and (**c**) 150 kGy. The curves correspond to an NO_2_ concentration of 10 ppm.

Figure 2a shows the resistance curve for the sensor fabricated from the pristine SnO_2_ nanowires (referred to as the pristine-nanowires sensor). Figure 2b,c are those of the sensors fabricated using the SnO_2_ nanowires irradiated with the electron beam at doses of 50 and 150 kGy, respectively (referred to as the 50-kGy-nanowires sensor and the 150-kGy-nanowires sensor, respectively). The resistance curves were determined over the course of three introduction/stoppage cycles of 10 ppm NO_2_.

In the undoped form, SnO_2_ is an *n*-type semiconductor^24^. The dominant species on the surfaces of SnO_2_ nanowires at 573 K is O^−^ 25,26. The resistance will be increased by the following reaction: O^−^ + V_O_^2+^ + e^−^ → O^O^. During the sensing experiments, the chemisorption of NO_2_ leads to the following reaction: NO_2_ (gas) + e^−^ → NO_2_^−^ 27,28. Electrons are extracted from the SnO_2_ surface, increasing the resistance of the nanowires. The NO_2_^−^ ions are dissociated into NO (g) and O^−^ by the following reaction: NO_2_^−^ → NO (g) + O^−^ 29, where the generated O^−^ ions contribute to a further increase in the resistance.

In the case of the pristine-nanowires sensor, the resistance increases sharply to a high value upon exposure to NO_2_ gas and then drops quickly to a low value upon the stoppage of the gas, resulting in a sensor response of 4.15 (Table 1). It is evident from Fig. 2a,b that the electron-beam irradiation caused a significant change in the sensing performance of the SnO_2_ nanowires. The 50-kGy-nanowires sensor continued to exhibit *n*-type behavior; this was because the irradiation dose was low (50 kGy), resulting in the irradiated SnO_2_ nanowires being similar to the pristine ones. However, the response of this sensor was estimated to be only approximately 1.02, indicating a significant degradation of the *n*-type sensing behavior. Additionally, the response time of the 50-kGy-nanowires sensor was as long as 277 s and much longer than that of the pristine-nanowires sensor, which was 28 s (Table 1). Furthermore, the recovery times of the pristine-nanowires and 50-kGy-nanowires sensors were 18 and 140 s, respectively. In case of the 150-kGy-nanowires sensor, the resistance decreases upon the introduction of the NO_2_ gas and increases on the subsequent stoppage of its flow. This unexpected behavior is indicative of *p*-type conductivity. The introduction of NO_2_ gas during the sensing experiments results in the extraction of electrons from the lattice. This further increases the number of positive holes, which are now the major carriers. The decrease in resistance owing to the increase in the number of hole carriers in a *p*-type semiconductor results in *p*-type sensing behavior. The response of the 150-kGy-nanowires sensor was estimated to be approximately 1.03. In addition, its response and recovery times were measured to be approximately 16 and 230 s, respectively. The Hall measurements confirm that the 150-kGy-nanowire is a p-type (Text S1 in Supplementary Information).

In order to investigate the origin of the *p*-type behavior observed in the 150-kGy-nanowires sensor, we performed XRD analyses and PL measurements. As mentioned previously, Fig. 1 shows that both the structural morphology and the surface roughness of the nanowires were not significantly affected by the electron-beam irradiation process. XRD spectra of the pristine and electron-beam-irradiated SnO_2_ nanowires are shown in Supplementary Information (Figure S2). Irrespective of whether the nanowires were irradiated or not, all recognizable reflection peaks present in the spectra could be indexed to a tetragonal rutile SnO_2_ phase (JCPDS card: No. 41-1445). The peak positions and full-width-at-half-maximum (FWHM) values of the (110), (101), and (211) diffraction peaks for the pristine and electron-beam-irradiated SnO_2_ nanowires are listed in Supplementary Information (Table S1). It can be seen from the table that FWHM values of the irradiated nanowires were not significantly different from those of the pristine ones (Figure S3). Furthermore, the positions of the main peaks in the XRD spectrum of the pristine nanowires did not change noticeably after the nanowires were irradiated. Accordingly, we conclude that not only the crystalline integrity but also the interplanar distance of the SnO_2_ structure remained unaffected by the electron-beam irradiation. Furthermore, by the examination of the XRD (Figure S4) and TEM data (Figure S5), the electron-beam irradiated SnO_2_ nanowires did not exhibit other phases, including tetragonal SnO.

By the electron-beam irradiation, the main PL emission peak at around 2.1 eV has been decreased (Figure S6, Text S2). With assumption that oxygen vacancies and tin interstitials are related to the yellow emission (~2.1 eV) in SnO_2_, it is possible that the electron-beam induced tin vacancies eliminate the tin interstitials or the electron-beam irradiation changed the characteristics of the oxygen vacancies.

Irradiation with an electron beam can generate holes by two mechanisms^30^. One possibility is that positively charged holes are generated via the following reaction: 1/2O_2(gas)_ = O_int_^*i*−^ + *ih*^30^,^31^. This is the anion-interstitial model, in which O_int_^−*i*^, *h*, *i* are an interstitial oxygen ion, the associated hole, and the degree of ionization (*i* = 1 or 2), respectively. The second possible mechanism is based on the cation-vacancy model, in which positively charged holes are generated by the following reaction: O_2(gas)_ = 2O_O_ + V_Sn_^*i*−^ + *ih*^32^ or Sn_Sn_ = V_Sn_^*i*−^ + *ih* +Sn_gas_, where V_Sn_^*i−*^ is the Sn vacancy and *i* = 1–4. On the basis of both the fact that the interstitial sites in the SnO_2_ lattice are much smaller than an oxygen ion in size and the results of the DFT calculations^2^, it can be surmised that the second reaction must be the main source of holes. That is, a strong electron beam will directly expel Sn atoms out of their lattice positions. Accordingly, mechanism related to the introduction of other defects contributing to *p*-type conduction can be exclusively disregarded. The expelled atomic tin is sublimated into air through the surfaces of the SnO_2_ nanowires, because tin cannot occupy the interstitial site, which will make SnO_2_ an *n*-type semiconductor. In this manner, the generated holes may play a greater role than the electrons at the surfaces of the nanowires, resulting in the observed *p*-type behavior in the SnO_2_ nanowires irradiated at a dose of 150 kGy. We calculate the concentrations for both carriers (electrons and holes) later in the paper.

**Figure 3:**
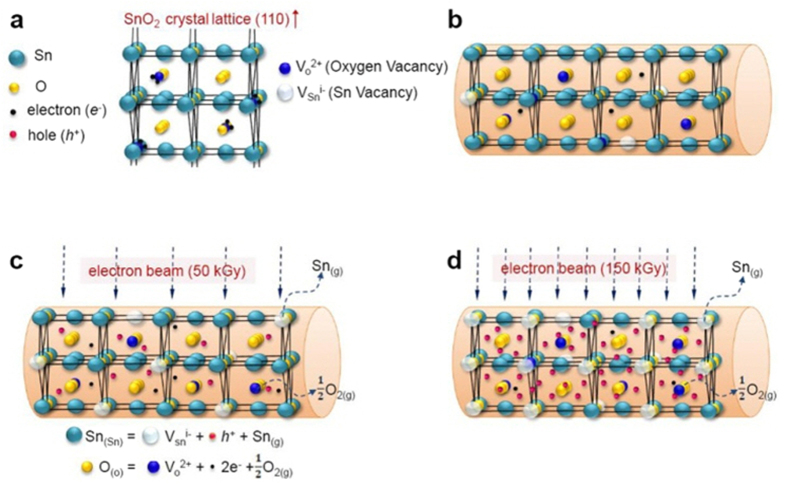
Schematics of electronic transport properties of e-beam-irradiated SnO_2_ nanowires. (**a**) A model of SnO_2_ crystal lattice. (**b**) Schematic diagram of the pristine SnO_2_ nanowires. Schematic outlines describing the effect of the electron-beam irradiation at doses of (**c**) 50 kGy and (**d**) 150 kGy.

We speculated the mechanisms on the changes induced by the electron-beam irradiation and showed the related schematic diagrams in Fig. 3. When the nanowires are irradiated with the electron beam, the beam electrons not only create oxygen vacancies but also tin vacancies which generate positive holes. The positive holes are attributed to the newly formed acceptors, otherwise they will be unstable due to their high formation energy. When a low irradiation dose (50 kGy) is used, positive holes are generated: Sn_Sn_ = V_Sn_^*i*−^ + *ih* + Sn_gas_ (Fig. 3c). In addition, negative electrons and oxygen vacancies are generated: O_o_ = V_o_^2+^ + 2 e^−^ + 1/2O_2gas_ (Fig. 3c). The overall conduction is still *n*-type due to insufficient amount of holes to match the increased oxygen vacancies. When a high irradiation dose (150 kGy) is used, a sufficient amount of positive holes are generated and positive holes become prevalent in the SnO_2_ lattice (Fig. 3d).

**Figure 4:**
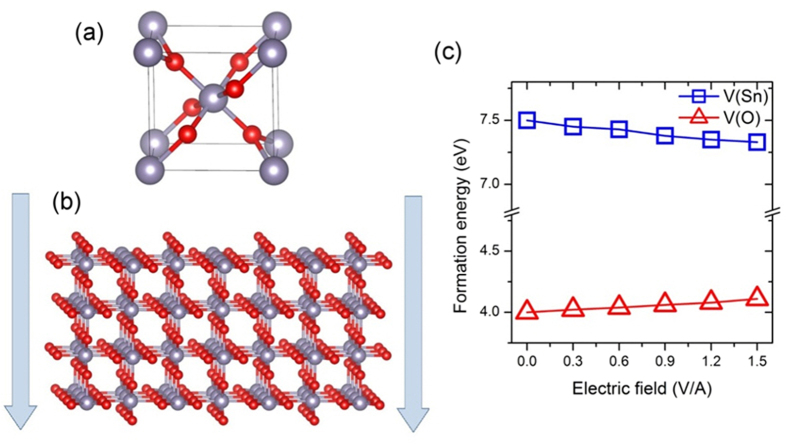
Crystal structures of SnO_2_ and formation energy of Sn and O vacancies. Crystal structure of (**a**) bulk SnO_2_ and (**b**) (110) thin film slab of SnO_2_. The vertical arrows indicate the direction of applied electric field. (**c**) Formation energy of Sn and O vacancies as a function of applied electric field.

To provide further insight into the origin of observed *p*-type behavior of SnO_2_ under e-beam radiation, the electronic properties of SnO_2_ nanostructures were calculated by density functional theory (DFT) methods. SnO_2_ has a rutile crystal structure in the space group *P*4_2_*/mnm* (No. 136) as shown in Fig. 4a. The atomic structure of SnO_2_ nanostructures thin film was initially constructed by cleaving the surface of the bulk structure of tin dioxide. Using this technique, it is possible to get nanostructures with a wide range of surface orientations and thicknesses while maintaining the bulk composition. The thickness of the studied SnO_2_ thin film is equal to 1.2 nm. During geometry optimization, all atoms are allowed to relax freely to their new equilibrium positions in order to obtain the lowest-energy configuration. The optimized structure is shown in Fig. 4b. Our analysis shows that among the bulk terminated low-index SnO_2_ surfaces (i.e. surfaces with surface-tin atoms in their bulk Sn^4+^ oxidation state), the (110) surface exhibits the lowest surface energy as compared to the (110) and (001) ones. This is in good agreement with experimental studies^33^. Therefore, we will only focus on the thin film slabs with (110) surface orientation in our study.

We then calculate the formation energy of a defect *D* in a charge state *q* as follows


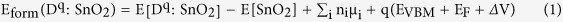


where 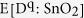 and 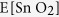 are the total energies of defected and perfect SnO_2_, respectively, 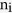 is the number of atoms being removed from the perfect system (*i* = Sn or O), 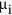 is the chemical potential. The last term describes the dependence on the Fermi level (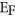) measured from the valence band edge, 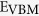 is a position of the valence band maximum (VBM) in the perfect system, and 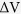 is a correction term aligning the VBM in the doped and perfect crystal supercells. Chemical potential 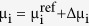 where 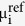 is calculated for the bulk Sn phase or O_2_ molecule, while 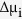 may vary corresponding to certain experimental growth conditions (*e.g.* Sn-rich/O-poor or Sn-poor/O-rich). Although PBE calculations typically underestimate band gaps of oxide semiconductors, they are proven efficient in predicting the general trends and defect formation energies^34^,^35^.

The effect of electron radiation on the system behavior was simulated by applying the external electric field, perpendicular to the SnO_2_ thin film surface. We place a single tin or oxygen vacancy in the core region nearby the top surface of SnO_2_ thin film. Under normal conditions (electric field is zero), the formation energies of neutral tin and oxygen vacancies are equal to 7.5 and 4.0 eV, respectively, at oxygen-rich limit. Note that at tin-rich limit, the formation energy of *V*_*O*_ defect decreases to 1.2 eV. These values are very close to their respective bulk values and consistent with literature data. Under both tin-rich and oxygen-rich conditions, the donor defects are dominant in the system. Very high formation energy of Sn vacancy indicates that its concentration would be negligible under equilibrium conditions. This explains why *p*-type conductivity is very difficult to achieve in experimental SnO_2_ samples.

**Figure 5:**
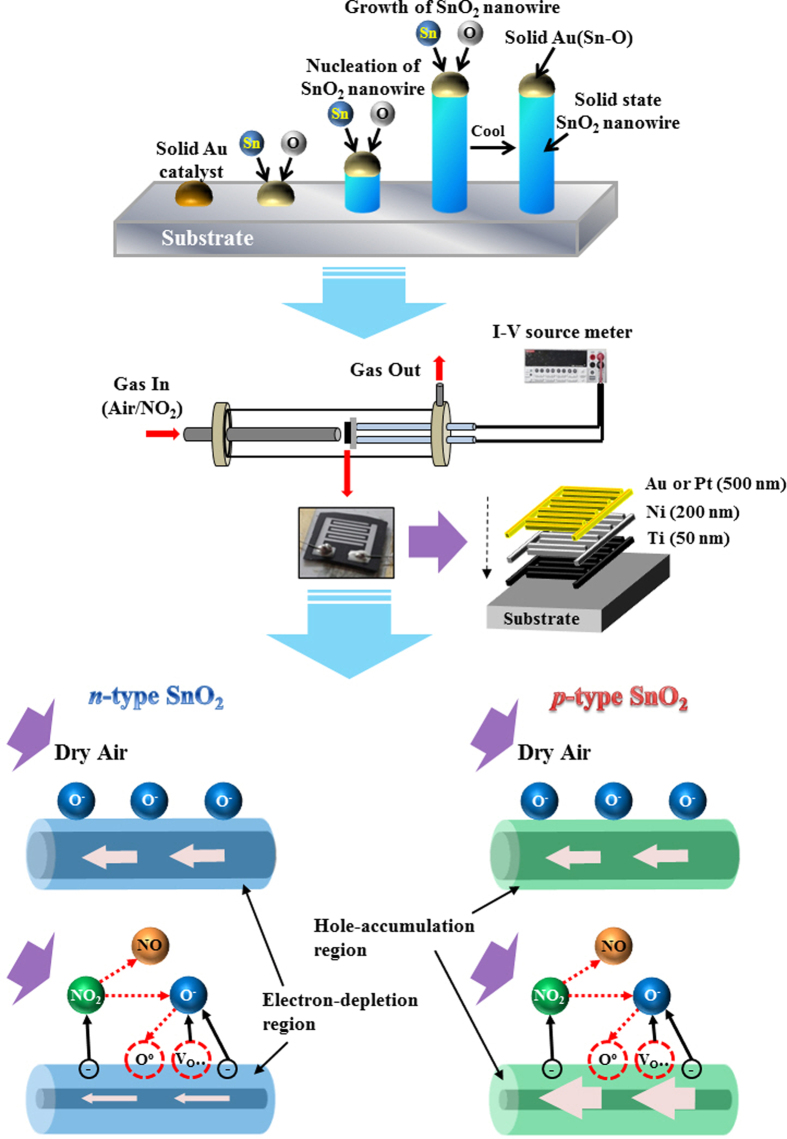
Preparation of sensor materials and devices. Schematic outline of preparation of sensor materials (i.e. SnO_2_ nanowires), fabrication of SnO_2_ nanowires gas sensors, and associated gas sensing mechanisms.

We next studied the effect of an external electric field on the defect properties of SnO_2_ nanostructures. Interestingly, the applied electric field has a substantial effect on the formation energies of both tin and oxygen vacancies. The calculated formation energies as a function of external electric field under oxygen-rich conditions (which should be more suitable for *p*-type defect formation) are shown in Fig. 5c. We notice that the formation energy of acceptor-type *V*_*Sn*_ defect decreases with the increase in electric field. This suggests that the formation of acceptor defects becomes more likely under the external electron radiation. This result can be rationalized as follows: the presence of external electric field also leads to the large polarization of SnO_2_ thin film with the formation of *p*-type-like region at the top surface of thin film and *n*-type region at the bottom. Inside the *p*-type-like zone at the surface of SnO_2_ nanostructure, the formation energy of acceptor V_Sn_ defects is slightly reduced.

In order to further investigate the origin of the observed *p*-type behavior, we measured the electrical resistances of the SnO_2_ nanowires after irradiation at different doses (Table 2). These values were obtained from the initial resistance, which were measured from the dynamic resistance curves in Fig. 2 of our manuscript (i.e. R_1_, R_2_, and R_3_ are 3000, 1245, and 935 Ω, for pristine (i.e., nonirradiated), 50-kGy-irradiated, and 150-kGy-irradiated SnO_2_ nanowires, respectively). The specific resistivity of pristine SnO_2_ (*n*-type) can be determined reasonably from basic semiconductor theory: specific resistivity *ρ* = 1/(*qμ*_*e*_*n*), where *q*, *μ*_*e*_, and *n* are the elementary charge of an electron, the mobility of the electrons, and their concentration, respectively. By assuming the values of *q* = 1.6 × 10^−19^ C, *μ*_*e*_ = 72 cm^2^/(V·s)^36^, and *n* = 3.3 × 10^16^ cm^−3^ 37, we obtain a specific resistivity of 2.63 Ω/cm. On the basis of the measured resistance of the pristine SnO_2_ nanowires and the specific resistivity of pristine SnO_2_, the equivalent length of the nanowires was calculated to be approximately 1140 cm. This was used to convert the measured resistance data into specific resistivity as shown in Table 2.

Next, we discuss in detail the electrical transport mechanism in the SnO_2_ nanowires under NO_2_ exposure, on the basis of standard hole gas equilibrium statistics. As shown in Table 2, the specific resistivities of the pristine, 50-kGy-irradiated, and 150-kGy-irradiated nanowires are designated as *ρ*_*1*_, *ρ*_*2*_, and *ρ*_*3*_, respectively. For the 50-kGy-irradiated nanowires,


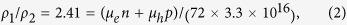


where *μ*_*e*_, *μ*_*h*_, *n*, and *p* are the electron mobility, hole mobility, electron concentration, and hole concentration, respectively. Accordingly, (*μ*_*e*_*n* + *μ*_*h*_*p*) = 5.73 × 10^18^ (cm·V·s)^−1^. In the present work, we set the following two extreme conditions:

(1) *μ*_*e*_*n* ≫ *μ*_*h*_*p* (that is, the nanowires are very similar to pristine SnO_2_ in nature)

(2) *μ*_*e*_*n* = *μ*_*h*_*p* (this corresponds to the exact point where the conduction behavior transitions from *n*- to *p*-type).

The hole mobility in SnO_2_ (which is lower than the electron mobility) is reported to be ~6 cm^2^/(V·s) in Mn-doped SnO_2_^38^ and ~39 cm^2^/(V·s) in In-Ga codoped SnO_2_^39^. In this study, we assume *μ*_*e*_ = 5 × *μ*_*h*_. The factor 5 is assigned on the basis of the effective mass ratio of a hole and an electron^40^,^41^. Therefore, we consider *μ*_*h*_ = 72/5 = 14.4 cm^2^/(V·s). This value falls between those measured previously^38^,^39^.

For condition 1, since *μ*_*e*_*n* = 5.73 × 10^18^ (cm·V·s)^−1^, the electron concentration is calculated to be approximately 8.0 × 10^16^ cm^−3^. For condition 2, *μ*_*e*_*n* + *μ*_*h*_*p* = 5.73 × 10^18^ (cm·V·s)^−1^, and thus the electron and hole concentrations are calculated to be approximately 4.0  × 10^16^ cm^−3^ and 2.0 × 10^17^ cm^−3^, respectively. Therefore, it is expected that electron and hole concentrations are in the ranges of 4.0 × 10^16^ to 8.0 × 10^17^ cm^−3^ and 0 to 2.0 × 10^17^ cm^−3^, respectively.

In the case of the 150-kGy-irradiated nanowires, the ratio of the specific resistivities is


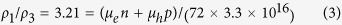


Then, *μ*_*e*_*n* + *μ*_*h*_*p* = 7.63 × 10^18^ (cm·V·s)^−1^. We again set two extreme conditions:

(1) *μ*_*e*_*n* ≪ *μ*_*h*_*p*

(2) *μ*_*e*_*n* = *μ*_*h*_*p*

For condition 1, since *μ*_*h*_*p* = 7.63 × 10^18^ (cm·V·s)^−1^, by assuming *μ*_*h*_ = 14.4 cm^2^/(V·s), the hole concentration is calculated to be approximately 5.3 × 10^17^ cm^−3^. For condition 2, *μ*_*e*_*n* + *μ*_*h*_*p* = 7.63 × 10^18^ (cm·V·s)^−1^, and thus the electron and hole concentrations are calculated to be approximately 5.3 × 10^16^ cm^−3^ and 2.7 × 10^17^ cm^−3^, respectively. Therefore, it is expected that the electron and hole concentrations are in the ranges of 0 to 5.3 × 10^16^ cm^−3^ and 2.7 × 10^17^ to 5.3 × 10^17^ cm^−3^, respectively (Table 2).

In the SnO_2_ nanowire system, we have two major carriers to work with: electrons and holes. By the electron-beam irradiation, tin vacancies, holes, oxygen vacancies, and electrons will be generated in regard to the following reactions: Sn_Sn_ = V_Sn_^*i*−^ + *ih* + Sn_gas_; Oo = V_o_^2+^ + 2 e^−^ + 1/2O_2gas_. Based on the DFT model, both oxygen vacancies and tin vacancies are located at near the conduction band edge. Accordingly, oxygen vacancies generate shallow donors, easily producing many electrons. On the other hand, tin vacancies are deep acceptors and they will produce negligible amount of holes in normal condition but can produce many holes by high-dose electron-irradiation. The response time is related to the physics on how long the minor carrier (or killers) is neutralized or deactivated, whereas the recovery time is linked to the physics on how long the major carrier is overtuned.

In case of pristine state without the irradiation (regime I), many electrons exist in conjunction with oxygen vacancies, whereas there are almost no holes and tin vacancies, as indicated by thermodynamics^42^. In this case, both the response and recovery times are dependent on the diffusion of oxygen vacancy. As shown in Table 1, the response and recovery times are 28 and 18 s, respectively. The appearance of very short response and recovery times will be related to the faster diffusion of oxygen vacancies than that of tin vacancies or tin atoms^43^,^44^. Furthermore, the response time (28 s) is longer than the recovery time (18 s), indicating that there is still very little tin vacancy-like defects which have to be deactivated in a slower process than the diffusion of oxygen vacancies.

In case of 50-kGy-irradiation (regime II), the response and recovery times increase to 277 and 140 s, respectively. Table 2 shows that both electron and hole concentrations increase by the 50-kGy-irradiation. Although there may be higher hole concentrations than electron concentrations, it will be still *n*-typed due to their difference in mobility. The initial step in the conduction process of the response step is the removal or deactivation of the deep acceptor (i.e. tin vacancy), which acts as a donor killer. Due to the slower diffusion of tin vacancies than that of oxygen vacancies, the elimination of tin vacancies takes much longer time than that of oxygen vacancies in the pristine case, resulting in the longer response time of 277 s for the case of 50-kGy irradiation. In other words, the increase of response time can be explained on the basis of the reduction in the number of electrons that can move freely without interacting with holes/donor killer, which were generated by the electron-beam irradiation. This generates additional energy barriers for the transport of electrons. Similarly, during the recovery step, the recovery of electrons (major carriers) is very fast (the same as in regime I), but the recovery of holes (minor) is no longer ignorable due to the considerable amount of holes to recover. In other words, the remaining or newly-formed tin vacancies have to be recovered (or deactivated), which is a slow process leading to a longer recovery time (140 s).

In case of 150-kGy-irradiation (regime III), the acceptor killer is an oxygen vacancy. Therefore, the fast diffusion of oxygen vacancies will result in a shorter response time (16 s). The long recovery time (230 s) is determined by the slow diffusion of tin vacancies. It is noteworthy that the recovery time of 150-kGy-irradiated sample (230 s) is slightly shorter than response time of 50-kGy-irradiated sample (277 s), in spite of the fact that they are operated by the same process: deactivation of tin vacancies. It may be due to the compensation effects of the minor carrier electrons. In this case, the net hole concentration is higher than the electron concentration in the pristine SnO_2_ nanowires, even after the difference in the mobility has been into consideration (i.e. *μ*_*e*_ = 5 × *μ*_*h*_).

In general, these gas response and recovery times are closely associated with catalysis on the surfaces of the nanowires; this process can be investigated using DFT calculations^45^,^46^,^47^. Therefore, in the near future, it should be possible to design and test chemical sensors directly using computer modeling and simulations.

The variation of resistivity by the electron-beam irradiation can be explained by means of the standard electron hole gas equilibrium statistics. In Table 2, for pristine SnO_2_ nanowires, the electron and hole concentrations are 3.3 × 10^16^ electrons cm^−3^ and 0 cm^−3^, respectively. For SnO_2_ nanowires irradiated at 50 kGy, we check two cases; (a) upper limit electrons/lower limit holes: in case of full recombination, there will be 8.0 × 10^16^ electrons cm^−3^. Obviously, this is *n*-typed and the electron concentration was increased by the electron-beam irradiation at 50kGy. (b) lower limit electrons/upper limit holes: there will be 2.0 × 10^17^ holes cm^−3^ and 4.0 × 10^16^ electrons cm^−3^. In case (b), considering that *μ*_*e*_ = 5 × *μ*_*h*_., n*μ*_*e*_ = p*μ*_*h*_. This matches with the sensor response data and specific resistivity data, indicating that the 50-kGy-irradiated sample is an *n*-type semiconductor. The specific resistivity data support that the resistance decreases by the electron-beam irradiation at 50 kGy. Accordingly, the electron concentration as an *n*-type will increase by the electron-beam irradiation at 50 kGy. For SnO_2_ nanowires irradiated at 150 kGy, we check two cases; (a) upper limit electrons/lower limit holes: there will be 2.7 × 10^17^ holes cm^−3^ and 5.3 × 10^16^ electrons cm^−3^, considering that *μ*_*e*_ = 5 × *μ*_*h*_., n*μ*_*e*_ < p*μ*_*h*_. (a) lower limit electrons/upper limit holes: there will be 5.3 × 10^17^ holes cm^−3^ and 0 electrons cm^-3^. This is obviously *p*-type semiconductor. Our model shows the increase of hole concentration (from tin vacancy) by increasing the dose from 50 kGy to 150 kGy. However, the 50-kGy- and 150-kGy-irradiated samples are *n*-typed and *p*-typed, respectively. We check two cases: (a) upper limit electrons/lower limit holes: from Table 2, 150-kGy-irradiated sample has (8-5.3) × 10^16^ less electrons (/cm^3^) than the 50-kGy-irradiated one, but the difference in hole concentration is 2.7 × 10^17^ (/cm^3^), which is sufficient to make up for the conduction loss due to the electron even after taking into the factor of 5 (difference in mobility between electron and hole). We can justify that 150-kGy-irradiated sample has a little lower resistivity than the 50-kGy-irradiated one. (b), lower limit electrons/upper limit holes: from Table 2, 150-kGy-irradiated sample has (5.3−2) × 10^17^ more holes (/cm^3^) than the 50-kGy-irradiated one. This difference is sufficient to make up for the shortage due to the (4.0-0) × 10^16^ electrons (/cm^3^) in 150-kGy-irradiated sample. Therefore, our model (and data in Table 2) justifies the slight lower resistivity in 150 KGy-irradiated sample.

## Discussion

Using the results of these calculations, we set the upper and lower limits for both carrier concentrations (Table 2), and explain the phenomena on experiment response and recovery times by a charge compensation mechanism. A typical charge compensation process on the Li- and Ti-codoping of NiO was studied by Wu *et al.*^48^; Li and Ti contributed to *p*-type and *n*-type conductions, respectively. On the basis of electron hole gas equilibrium statistics, the dependence of the Li-and Ti-generated states can be divided into three regimes: I, II, and III, as shown in Fig. 2 of Ref.^46^. The regimes I, II, and III correspond to the donor-dominated, compensated, and acceptor-dominated states, respectively. In the present work, transport behaviors associated with these three regimes were observed in zero, 50-, and 150-kGy-irradiated SnO_2,_ where shallow donor and deep acceptor states are formed by oxygen vacancies and tin vacancies, respectively. In regime I, electrons are the only carriers. In regime II, electrons and holes are the major and minor carriers, respectively, leading to an n-type conduction. In regime III, holes and electrons are the major and minor carriers, respectively, leading to a p-type conduction.

Upon NO_2_ exposure, the concentrations of oxygen and tin vacancies will decrease and increase, respectively, and are responsible for the measured resistivity changes. The response time for resistivity change depends on the mobility of the minor carriers (non, holes and electrons respectively for regime I, II and III, respectively), via the charge compensation mechanism. There is negligible minority carrier in regime I and the minority carriers for regimes II and III are holes and electrons, respectively. For instance in regime III, newly generated holes (by NO_2_ exposure) have to first compensate the donor states (minor carriers) before contributing to the noticeable decrease in resistivity. Similarly the recovery time depends on the mobility of the major carriers, which have to return to the original states. In SnO_2_, it is believed that tin vacancies (for holes) diffuse slower than oxygen vacancies (for electrons)^43^. Therefore, it predicts a prolonged response time if holes are the minor carriers and an extended recovery time if the holes are the major carriers.

In summary, we were able to achieve *p*-type-like conduction behavior in initially *n*-type SnO_2_ nanowires via irradiation with a high-energy electron beam. We have proposed a mechanism for the transition from *n*-type to *p*-type conduction after electron-beam irradiation at a high dose (150 kGy). We explain that the electron-beam-induced loss of tin atoms (i.e. the generation of tin vacancies) in the SnO_2_ lattice generates corresponding holes in the valence band of SnO_2_, eventually leading to the *p*-type behavior. The concentrations of both carriers (electrons and holes) in the SnO_2_ nanowires estimated on the basis of standard hole gas equilibrium statistics could explain the transition from *n*- to *p*-type transport behavior after the irradiation. The accompanying changes in the NO_2_-sensing performances of the nanowires, including those in the response and recovery times, could also be explained. This study not only demonstrates that electron-beam irradiation can be an effective means of creating bipolar transport conduction in semiconductors but also provides a fundamental approach for developing SnO_2_-based electronic and electrochemical devices.

## Methods

### E-beam irradiation

The SnO_2_ nanowires were fabricated by heating Sn powders via a vapor-liquid-solid (VLS) method (Fig. 5). The substrate temperature was kept at 800 °C, while a mixture of Ar and O_2_ gases was made to flow through the growth chamber. The fabrication procedure used was similar to one previously reported^49^. Once fabricated, the SnO_2_ nanowires were irradiated with an electron beam. The apparatus used for the process was the ELV-8 electron accelerator (EBTech, Daejeon, Korea). Electron beams with an accelerating voltage, dose, beam current, and pulse duration of 1.5 MeV, 50–150 kGy, 1 mA, and 400 ps, respectively, were used. The KGy is equal to 1,000 Gy, and the Gy is defined as the absorption of one joule of ionizing radiation by one kilogram of matter. The synthesized SnO_2_ nanowires were irradiated in air ambient at room temperature. For precise comparison, the pristine and electron-beam irradiated samples not only are the same size but also contained nearly the same amount of SnO_2_ nanowires within the error range, being confirmed by the analytical balance (readability: 0.01 mg, XR 205SM-DR, PreCISA Instruments AG, Swiss).

### Characterization

The phases and crystalline qualities of the nanowires were determined via X-ray diffraction (XRD) analyses performed using a Philips X’pert MRD diffractometer and CuKα_1_ radiation. The microstructures of the nanowires were observed using field-emission scanning electron microscopy (FE-SEM) (Hitachi S-4200). In order to investigate the nature of the defects in the nanowires, the photoluminescence (PL) spectra of the nanowires were recorded at room temperature using a SPEC-1403 photoluminescence spectrometer and the 325-nm line of an He-Cd laser (Kimon, Japan).

### Sensing measurement

For the NO_2_ sensing measurements, an electrode layer comprising a 300-nm-thick layer of Au and a 50-nm-thick layer of Ti was sequentially sputtered on the specimens with an interdigital electrode mask (Fig. 5). A preliminary study performed by us had indicated that this Ti/Au system is very effective for making the ohmic contacts for the sensors. The fabricated sensors were placed in a horizontal-type tube furnace; the operating temperature was fixed at 300 °C, because the NO_2_-sensing performances of the sensors were the best at this temperature during preliminary tests. The concentration of NO_2_ was controlled by changing the mixing ratio of dry air to NO_2_ gas using high-precision mass flow controllers. The total flow rate was set to 500 sccm to prevent any possible variation in the sensing properties of the nanowires. According to the specifications provided by the gas manufacturer (Daeduk Gas Co., Korea), the water content in both the dry air/NO_2_ gas mixture and the dry air was negligible. In case of the sensor showing *n*-type sensing behavior, the sensor response (*R*) was determined using the following formula: *R* = *R*_*g*_/*R*_*a*_, where *R*_*a*_ and *R*_*g*_ are the resistances of the sensor in the absence and presence of NO_2_, respectively. In case of the sensor showing *p*-type sensing behavior, the response was determined using *R* = *R*_*a*_/*R*_*g*_. The response and recovery times of the sensors were defined as the times taken for the resistance to change by 90% on exposure to the target gas (NO_2_) and air, respectively.

### DFT studies

Our spin-polarized calculations were performed using Quantum Espresso package^50^. The exchange-correlation potential is approximated by generalized gradient approximation (GGA) using Perdew-Burke-Ernzerhof (PBE) functional^51^. The kinetic-energy cutoffs for valence electron wave functions and charge density are set as 35 Ry and 350 Ry, respectively. The optimized structures are obtained by relaxing all atomic positions using the Broyden-Fletcher-Goldfarb-Shanno (BFGS) quasi-Newton algorithm until the interatomic forces are less than 0.01 eV Å^−1^.

## Additional Information

**How to cite this article**: Kim, S. S. *et al.* Promotion of acceptor formation in SnO_2_ nanowires by e-beam bombardment and impacts to sensor application. *Sci. Rep.* **5**, 10723; doi: 10.1038/srep10723 (2015).

## Supplementary Material

Supplementary Information

## Figures and Tables

**Table 1:** Responses, response times, and recovery times of the sensors fabricated using the pristine and e-beam-irradiated SnO_2_ nanowires.

	**Pristine-nanowires sensor**	**50-kGy-nanowires sensor**	**150-kGy-nanowires sensor**
Response	4.15	1.02	1.03
Response time (s)	28	277	16
Recovery time (s)	18	140	230

**Table 2:** Resistances, specific resistivities, and upper and lower electron and hole concentrations of the pristine and e-beam-irradiated SnO_2_ nanowires.

	**Pristine SnO_2_ nanowires**	**SnO_2_ nanowires irradiated at 50 kGy**	**SnO_2_ nanowires irradiated at 150 kGy**
Resistance (Ω)	R_1_ = 3000	R_2_ = 1245	R_3_ = 935
Specific resistivity (Ω/cm)	ρ_1_ = 2.63	ρ_2_ = 1.09	ρ_3_ = 0.82
Upper limit of electron concentration (cm^−3^)	3.3 × 10^16^	8.0 × 10^16^	5.3 × 10^16^
Lower limit of hole concentration (cm^−3^)	0	0	2.7 × 10^17^
Lower limit of electron concentration (cm^−3^)	3.3 × 10^16^	4.0 × 10^16^	0
Upper limit of hole concentration (cm^−3^)	0	2.0 × 10^17^	5.3 × 10^17^
