# Supplementary material for: Promotion of acceptor formation in SnO2 nanowires by e-beam bombardment and impacts to sensor application
Source: Sci Rep. 2015 Jun 1;5:10723. doi: 10.1038/srep10723 (PMC5377233; doi:10.1038/srep10723)
Supplement: Supplementary Information [file srep10723-s1.doc]

**SUPPLEMENTARY INFORMATION**

**Promotion of acceptor formation in SnO_2_ nanowires by e-beam bombardment and impacts to sensor application**

Sang Sub Kim^1^, Han Gil Na^2^, Hyoun Woo Kim^2^,*, Vadym Kulish^3^, and Ping Wu^3^,*

^1^Department of Materials Science and Engineering, Inha University, Incheon 402-751, Republic of Korea.

^2^Division of Materials Science and Engineering, Hanyang University, Seoul 133-791, Republic of Korea.

^3^Entropic Interface Group, Singapore University of Technology & Design, Singapore 138682, Singapore.

Correspondence and requests for materials should be addressed to H. W. K. ([hyounwoo@hanyang.ac.kr](mailto:hyounwoo@hanyang.ac.kr)) and P. W. ([wuping@sutd.edu.sg](mailto:wuping@sutd.edu.sg)).


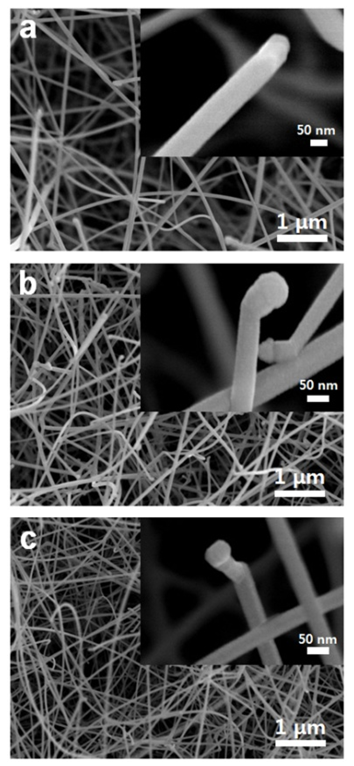


**Supplementary Figure S1** │ Scanning electron microscope (SEM) images of the (a) pristine SnO_2_ nanowires and those irradiated with the electron beam at doses of (b) 50 kGy and (c) 150 kGy. Insets are the corresponding high-magnification of nanowire tips.


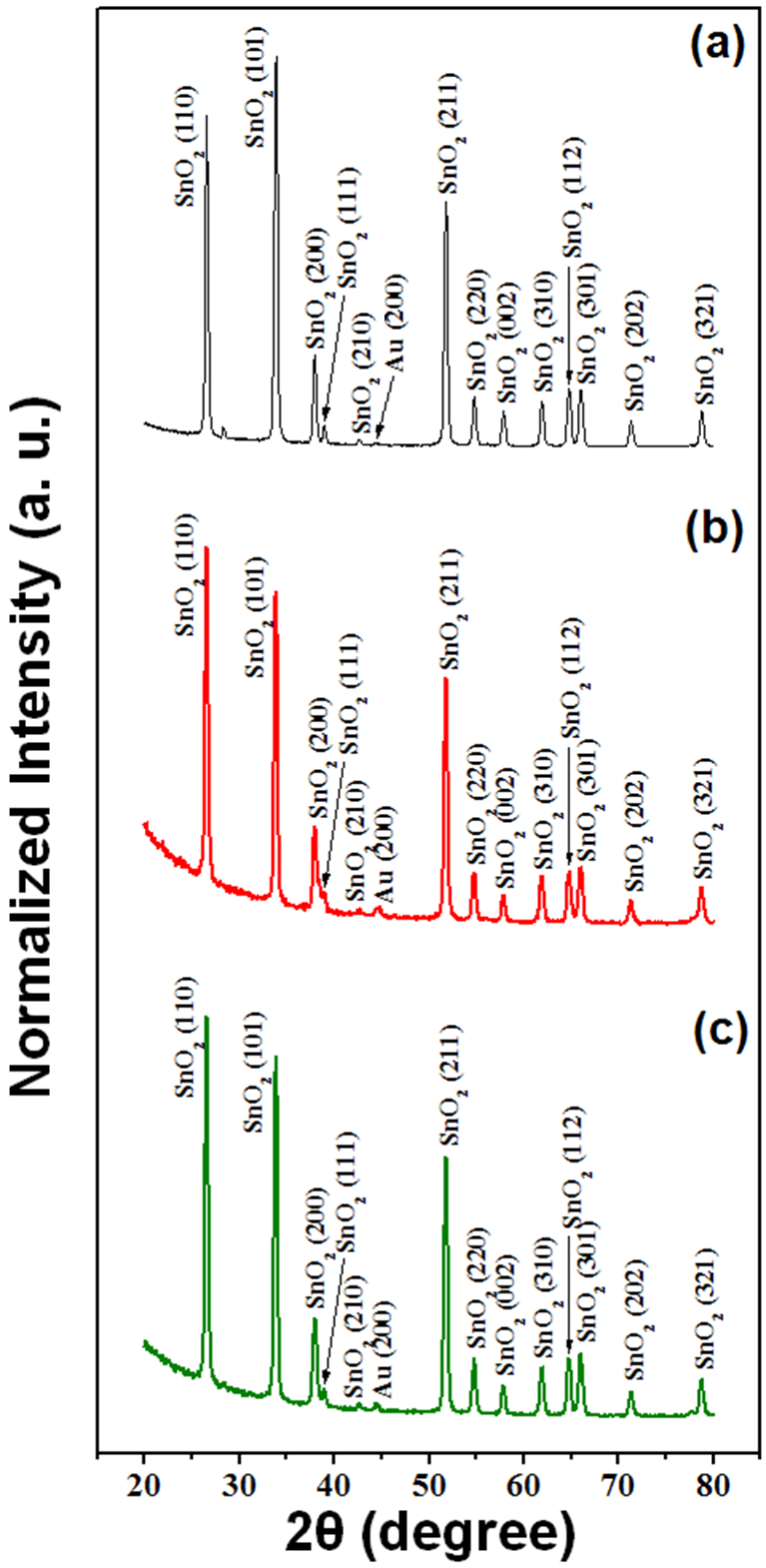


**Supplementary Figure S2** │ XRD spectra of the (a) pristine SnO_2_ nanowires and nanowires irradiated at doses of (b) 50 kGy and (c) 150 kGy.


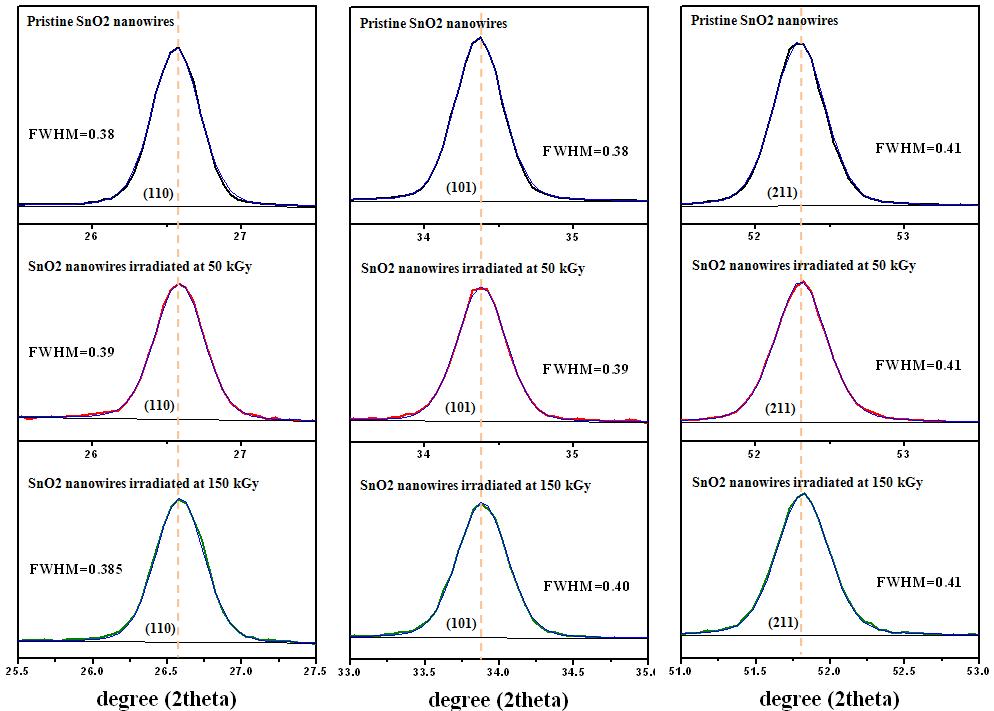


**Supplementary Figure S3** │ Peak profiles used for evaluating the FWHM values and monitoring the peak values.


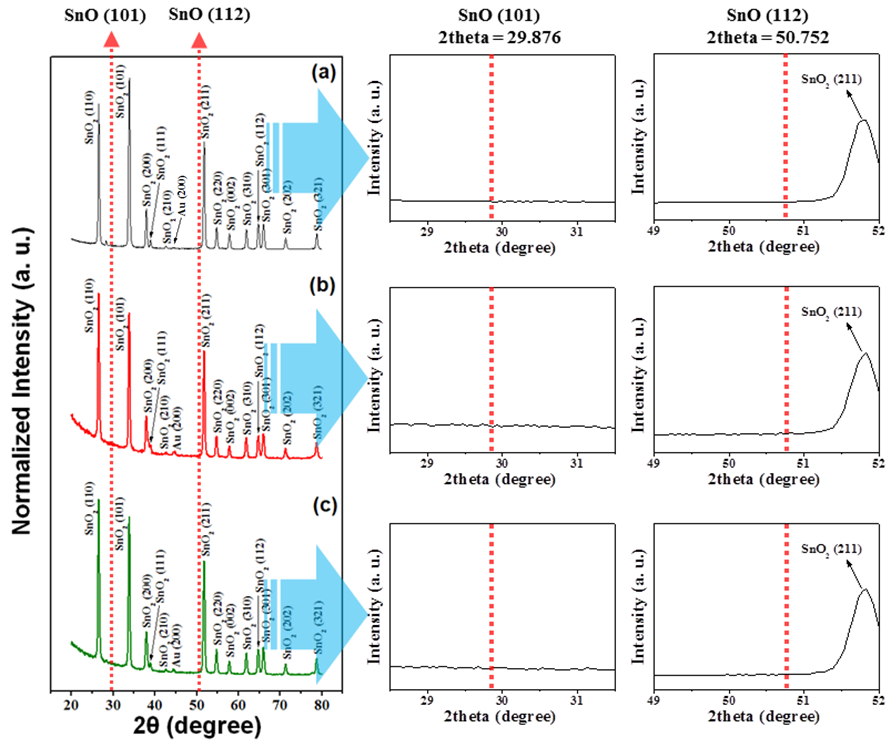


**Supplementary Figure S4** │ Examination of the possible existence of SnO(101) and SnO(112) peaks in the XRD spectra of the (a) pristine SnO_2_ nanowires and nanowires irradiated at doses of (b) 50 kGy and (c) 150 kGy.


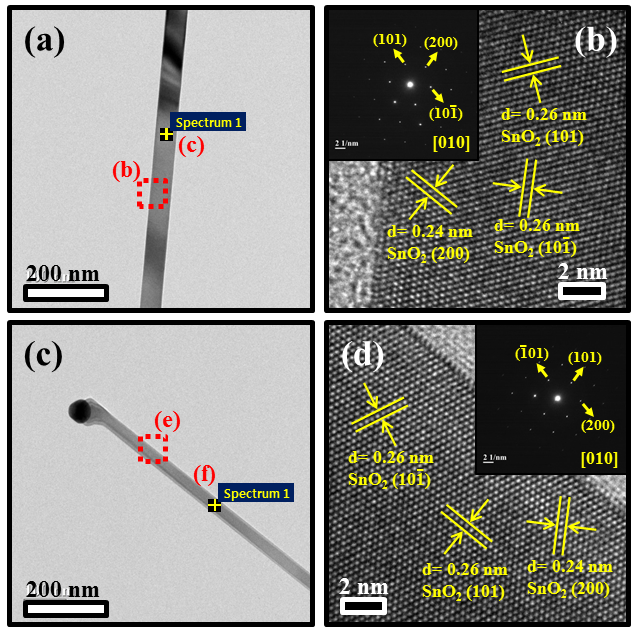


**Supplementary Figure S5** │ **TEM analysis of SnO_2_ nanowires, which are electron-beam irradiated at (a,b) 50 KGy and (c,d) 150 kGy.** (a,c) TEM images of electron-beam irradiated SnO_2_ nanowires. (b,d) Lattice-resolved TEM images and SAED patterns.


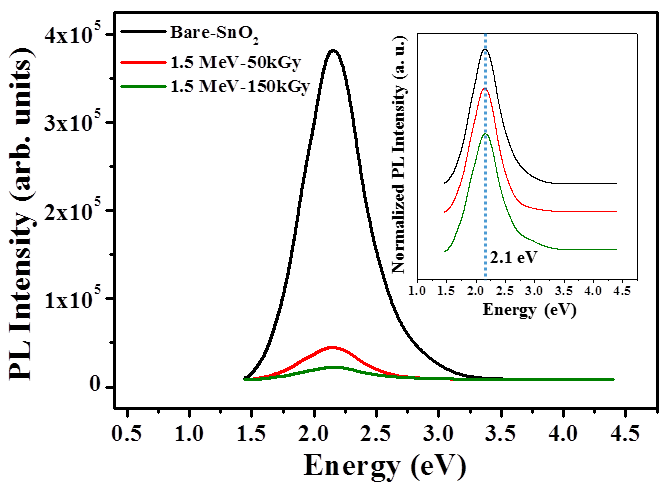


**Supplementary Figure S6**│ As-measured PL spectra of the pristine SnO_2_ nanowires and of those irradiated at doses of 50 kGy and 150 kGy. (Upper-right insets: normalized PL spectra).

|  | **Pristine SnO_2_ nanowires** | | **SnO_2_ nanowires**  **irradiated at 50 kGy** | | **SnO_2_ nanowires**  **irradiated at 150 kGy** | |
| --- | --- | --- | --- | --- | --- | --- |
|  | **Peak**  **(degree)** | **FWHM**  **(degree)** | **Peak**  **(degree)** | **FWHM**  **(degree)** | **Peak**  **(degree)** | **FWHM**  **(degree)** |
| (110) | 26.5592 | 0.380 | 26.5829 | 0.390 | 26.5850 | 0.385 |
| (101) | 33.8650 | 0.380 | 33.8827 | 0.387 | 33.8850 | 0.400 |
| (211) | 51.7950 | 0.410 | 51.8250 | 0.400 | 51.8150 | 0.410 |

**Supplementary Table S1** **│** Positions and FWHM values of the (110), (101), and (211) diffraction peaks of the pristine and e-beam-irradiated SnO_2_ nanowires.

**Text S1. Hall measurements**

We determined the carrier type of pristine and irradiated (150 kGy) SnO_2_ nanowires using the van der Pauw geometry and Hall effect measurements. The Hall effect measurements were performed at room temperature in air using Ecopia’s HMS-3000 Hall effect measurement system with a 1.0 T magnet. The carrier concentration was determined using the following equations^1^:


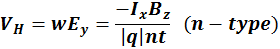
,

(1)


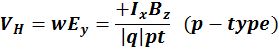
 .

Since V_H_, B_Z_, I_X_, t, and q are known (by measurement), it is possible to solve for the carrier concentration, n or p, and determine whether the sample is n-type or p-type. The exact thickness of the nanowire film, w, could not be evaluated in this experiment for several reasons. First, the films were comprised of irregularly-shaped nanowires with significant gaps, so the determination of w values corresponding to dense films was not possible. Second, during measurements, 4 electrodes are pressed to the nanowire films, so their depth could not be evaluated. Third, the thickness of the nanowire films was not uniform. Accordingly, we set the value of w to 3 μm, because we were interested in determining the primary carrier type of the nanowires. The measurements were repeated five times. For pristine nanowires, the carrier concentrations were calculated to be -4.3 x 10^17^, -5.0 x 10^17^, -3.4 x 10^17^, -1.2 x 10^17^, and -6.6 x 10^17^ cm-^3^, with an average value of -4.1 x 10^17^ cm-^3^. For the 150-kGy-nanowires, the carrier concentrations were calculated to be 3.1 x 10^13^, 2.8 x 10^12^, 2.0 x 10^13^, 4.4 x 10^12^, and 1.6 x 10^13^ cm-^3^, with an average value of 1.5 x 10^13^ cm-^3^. Accordingly, pristine and 150 kGy-electron-beam-irradiated nanowires were determined to be n-type and p-type, respectively.

**Text S2. PL analysis**

Figure S6 shows the PL spectra of pristine and electron-beam-irradiated SnO_2_ nanowires. The relative spectral peak intensities of the pristine, 50-kGy-irradiated, and 150-kGy-irradiated SnO_2_ nanowires were calculated to be approximately 17.3, 2.0, and 1.0, respectively. Accordingly, the overall PL intensity of the nanowires was reduced considerably by the electron-beam irradiation; irradiation at higher doses reduced the PL intensity to a greater degree. The inset shows the normalized PL spectra of the SnO_2_ nanowires. All spectra are composed primarily of a yellow emission band, which is centered at approximately 2.1 eV. Although the shape of the spectrum did not vary significantly after 50 kGy irradiation, the intensity was significantly reduced. It has been generally accepted that oxygen vacancies and tin interstitials are related to the yellow emission (~2.1 eV) in SnO_2_.^2,3^ One possibility is that tin interstitials are decreased by the electron-beam irradiation, in which the electron-beam-induced tin vacancies combine with tin interstitials, or beam electrons may activate and expel the tin interstitials to the surrounding environment. A second possibility is that the electron-beam irradiation changes the nature of the oxygen vacancies.^4^ The predominantly 100°-coordinated oxygen vacancies exist under normal circumstances, which are responsible for the emission peak observed near 2.1 eV. Electron-beam irradiation results in local heating and the 100°-coordinated oxygen vacancies then change into other types of oxygen vacancies, depressing the 2.1-eV emission. At a sufficiently high temperature, they change to 130°-coordinated oxygen vacancies, which are associated with high-energy PL emission.^4^ Although the electron-beam irradiation will generate oxygen vacancies, most will be converted to other forms of oxygen vacancies that do not contribute to yellow emission.

**Supplementary References**

1. Seki, S., Saeki, A., Sakurai, T. & Sakamaki, D. Charge carrier mobility in organic molecular materials probed by electromagnetic waves. *Physical Chemistry Chemical Physics.* **16**, 11093-11113 (2014).
2. Yamazoe, N. & Shimanoe, K. Theory of power laws for semiconductor gas sensors. *Sens. Actuators B* **128**, 566-573 (2008).
3. Ruhland, B., Becker Th. & Müller, G. Gas-kinetic interactions of nitrous oxides with SnO_2_ surfaces. *Sens. Actuators B* **50**, 85-94 (1998).
4. Wang, S. H., Chou T. C. & Liu, C. C. Nano-crystalline tungsten oxide NO_2_ sensor. *Sens. Actuators B* **94**, 343-351 (2003).
